# Histological confinement of transglutaminase-mediated nit sheath crosslinking is essential for proper oviposition and egg coating in the human head louse, Pediculus humanus capitis

**DOI:** 10.21203/rs.3.rs-2559266/v1

**Published:** 2023-02-09

**Authors:** Ju Hyeon Kim, Do Eun Lee, SangYoun Park, John M. Clark, Si Hyeock Lee

**Affiliations:** Seoul National University College of Medicine; Seoul National University; Soongsil University; University of Massachusetts; Seoul National University

## Abstract

**Background:**

Head louse females secrete liquid gel, which is mainly composed of the louse nit sheath protein 1 (LNSP1) and LNSP2, when they lay eggs. The gel is crosslinked by transglutaminase (TG) to form the nit sheath, which covers most part of egg except the top operculum area where breathing holes are located. Knowledge on the selective mechanism of nit sheath solidification to avoid uncontrolled crosslinking could lead to design a novel way of louse control, but no information is available yet.

**Methods:**

To elucidate the crosslinking mechanisms of nit sheath gel inside the reproductive system of head louse females, *in situ* hybridization in conjunction with microscopic observation of the oviposition process was conducted.

**Results:**

Histochemical analysis revealed that *LNSP1* and *LNSP2* are expressed over the entire area of accessory gland and uterus, whereas TG expression site is confined to a highly localized area around the opening of posterior oviduct. Detailed microscopic observations of oviposition process uncovered that a mature egg is positioned in the uterus after ovulation. Once aligned inside the uterus, the mature egg is redirected so that its operculum side tightly held by the ventral end of uterus being positioned toward the head again and its pointed bottom end being positioned toward the dorsal end of uterus, which functions as a reservoir for the nit sheath gel.

**Conclusions:**

Physical separation of the TG-mediated crosslinking site from the ventral end of uterus is necessary to avoid uncontrolled crosslinking inside the uterus and to ensure selective crosslinking over only the lower part of egg without any unwanted crosslinking over the operculum during oviposition.

## Background

Both head lice (*Pediculus humanus capitis*) and body lice (*Pediculus humanus humanus*) are obligatory ectoparasites, exclusively feeding on human blood. Human head lice cause economic and social problems worldwide, whereas human body lice pose a serious public health threat by transmitting several bacterial diseases [1]. Human louse females secrete liquid gel (louse glue), which later forms a protective egg covering, called nit sheath, to attach newly laid eggs to hair or fabrics. The nit sheath gel is produced from a pair of large female accessory glands and secreted into the uterus. For oviposition, a female louse secretes the gel onto the hair shaft first, spreads it using the last abdominal segment and then lays an egg, with the pointed (bottom) end of the egg being released first along with the gel [2]. The gel secretion diminishes before the anterior operculum side of the egg is excluded, which likely contributes to the protection of the operculum, a lid-like structure where aeropyles for gas exchange are located and through which the 1st instar nymph hatches, from being occluded by the liquid gel [3]. Following oviposition, the nit sheath gel coated over mostly the bottom part of the egg is completely solidified to form the nit sheath, thereby attaching it to the hair [4].

By analyzing the amino acid composition of the nit sheath proteins, two homologous proteins, named as louse nit sheath protein (LNSP) 1 and LNSP2, were identified [5]. More recently, LNSP1 and LNSP2 along with two hypothetical proteins were confirmed to be the major structural proteins through analyzing the transcriptome of the accessory gland plus uterus [6]. Using RNA interference (RNAi), LNSP1 and LNSP2 were determined to function in desiccation resistance and lubrication, thereby ensuring normal oviposition and embryo survival. In addition to LNSP1 and LNSP2, several proteins including transglutaminase (TG), defensin 1 and defensin 2, were identified to have essential functions. Knockdown of *TG* also impaired egg hatching, suggesting its role in the crosslinking of nit sheath protein. The role of TG in crosslinking was further confirmed by the treatment of GGsTop, a TG inhibitor. Taken together, controlled crosslinking of nit sheath gel, mainly composed of LNSP1 and LNSP2, is essential for producing functional nit sheath that ensures egg viability besides their function as glue.

Uncontrolled rapid solidification of the nit sheath gel before the complete exclusion of an egg can be fatal to a female as the incompletely excluded egg sticks to the vagina and blocks oviposition. Sometimes, the female ovipositor is observed to be strongly stuck to a hair by abnormally solidified nit sheath gel, resulting in eventual death of the female during oviposition, as described previously [3]. This phenomenon was particularly observed in old females (JHK, personal observation). On the other hand, retarded solidification is also detrimental to egg development, as previously demonstrated by impairing the nit sheath gel crosslinking via GGsTop [6]. Since both LNSP1 and LNSP2 are exclusively expressed in the accessory gland [6], it is necessary to precisely regulate the crosslinking of nit sheath gel to occur not inside the accessory glands but inside the uterus only when a mature egg is housed. In addition, a specific mechanism is required to prevent the nit sheath covering and solidification over the operculum area. However, it is unknown yet how human lice avoid the uncontrolled crosslinking inside the reproductive system and regulate selective crosslinking of nit sheath only over the bottom part of the egg.

In this study, therefore, in order to elucidate the crosslinking mechanisms of nit sheath gel inside the female reproductive system, *in situ* hybridization in conjunction with microscopic observation of the oviposition process was conducted. Detailed oviposition processes were constituted from multiple observations of dissected gravid females. A sophisticated mechanism for avoiding uncontrolled crosslinking inside the uterus and the selective crosslinking over the only lower part of egg during oviposition was proposed. Since the controlled formation of the egg sheath is very crucial for both female and embryo survival, this step can be exploited as a potential target for louse control.

## Methods

### Lice rearing

The South Florida strain of human head lice (SF-HL. *Pediculus humanus capitis)* have been reared on the *in vitro* membrane feeding system [7] under environmental conditions of 30°C and 70% relative humidity and 16/8 h light/dark in a rearing chamber (reviewed and approved by the Institutional Review Board of Seoul National University, IRB No. E1911/003–016).

### Probe synthesis

Total RNA was extracted from 5-day-old female lice with TRI reagent (MRC, Cincinnati, OH, USA) and treated with DNase I (Takara Biotechnology, Shiga, Japan) according to the manufacturer’s protocol. First-strand cDNA was synthesized using SuperScript IV reverse transcriptase (Invitrogen, Carlsbad, CA, USA).

For probe synthesis, *LNSP1, LNSP2* and *TG* fragments were amplified from the female cDNA (primer sequences are shown in Additional file 1: Table S1). For *LNSP1* and LNSP2 that show high sequence similarities, respective probe was designed from gene-specific sites in the N terminal domains. The PCR products were cloned into pGEM-T easy vector (Promega, Madison, WI, USA). Each plasmids were digested by ApaI (New England Biolabs, Ipswich, MA, USA) for sense probe (negative control) or NdeI (New England Biolabs) for antisense probe at 37°C for 1 h. The plasmids were checked by electrophoresis to confirm digestion and purified using Monarch^®^ PCR & DNA Cleanup Kit (New England Biolabs). Sense or antisense *LNSP1, LNSP2* and *TG* probes were generated using T7 or SP6 RNA polymerase (Promega). FITC RNA labeling mix or DIG RNA labeling mix (both from Roche, Mannheim, Germany) were used for *LNSP1/LNSP2* probe or *TG* probes, respectively.

#### in situ hybridization

Accessory glands and uterus were dissected from 5-day-old females in ice-cold RNase-free PBS (pH 7.4) (for reproductive system of human head louse, see additional file 2: Fig. S1). Tissues were incubated in 0.01% collagenase (Sigma) in PBS for 1 min with gentle rocking to improve the penetration of probe and reagents. After washing three times with PBS, tissues were fixed in 4% paraformaldehyde at 4°C overnight. Tissues were washed with PBS, followed by dehydration and rehydration using a series of ethanol baths. Hybridization was conducted in hybridization solution containing *LNSP1, LNSP2* or *TG* probes for 20 h at 58°C. After washing three times each with 5X and 0.2X SSC buffers at 63°C, subsequent protocols were adjusted depending on the experiments. Tissues for *LNSP1* and *LNSP2* were washed with PBST and then mounted on a glass slide with Vectashield (Vector Laboratories, Burlingame, CA, USA) for further confocal microscopy (SP8 X STED confocal microscope, Leica). In case of *TG* experiment, tissues were incubated with anti-DIG-AP Fab fragments (Roche) in a blocking reagent at 4°C overnight. The hybridization signal was visualized by treatment of NBT and BCIP (Roche) in NTMT buffer.

### Examination of oviposition process

The abdomens of 5-day-old gravid females were dissected in PBS under a stereo microscope (Greenough stereo microscopes S9i, Leica), and the oviposition stage of each dissected female was determined. Based on the observation that a 5-day-old female lays approximately five eggs a day, the time required for a single egg oviposition was assumed to be ~ 290 min. After dissecting a total of 75 females, the duration of each stage was roughly estimated by following calculation: (Total number of observed oviposition stage/75) · 290 (min).

## Results

### Histological characterization of TG-mediated crosslinking inside the uterus

**Figure 1:**
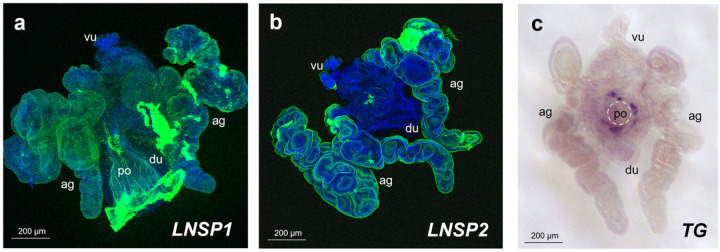
Histological sites of *LNSP1, LNSP2* or *TG* transcription in the accessory glands and uterus. Representative microscopic images of the accessory glands and uterus of head louse females following *in situ* hybridization with *LNSP1* (a), *LNSP2* (b) or *TG* (c) probe. **a, b** FITC-labeled probes were used for *LNSP1* and *LNSP2*. Both *LNSP1* and LNSP2were mainly detected in the entire areas of accessory gland with reduced expression being detected in the uterus (green signal). Images of nuclear staining (blue signal) and *LNSP1* or *LNSP2* were merged. **c**For *TG,* DIG-labeled probes were used for signal amplification. TGwas exclusively expressed at the focal area around the opening of posterior oviduct (dark purple). A dotted circle indicates the opening of posterior oviduct where an egg passes through for oviposition. ag, accessory gland; vu, ventral end of uterus; du, dorsal end of uterus; po, posterior oviduct.

To elucidate where and when the crosslinking occurs, histological properties of LNSP1, LNSP2 and TG were investigated. As expected, positive signals of both *LNSP1* and *LNSP2* were mainly detected over the entire area of accessory gland with reduced expression being detected in the uterus (Fig. 1a, b; for the negative control (sense probe) images, see additional file 3: Fig. S2). Although two morphologically distinguishable lobes were present in each side accessory gland, no apparent differences in the signals of *LNSP1* or *LNSP2* were observed between the two lobes, suggesting that both LNSP1 and LNSP2 are expressed in the same tissues. In contrast, *TG* signal was exclusively detected in a highly localized area around the opening of posterior oviduct (Fig. 1c; see Supplementary Fig. S2 for the negative control). These results strongly demonstrated that large amounts of both LNSP1 and LNSP2 are commonly expressed in the accessory glands and uterus without any histological separation, whereas TG expression is limited to a very narrow area around the opening of posterior oviduct.

### Microscopic observation of oviposition process

**Figure 2:**
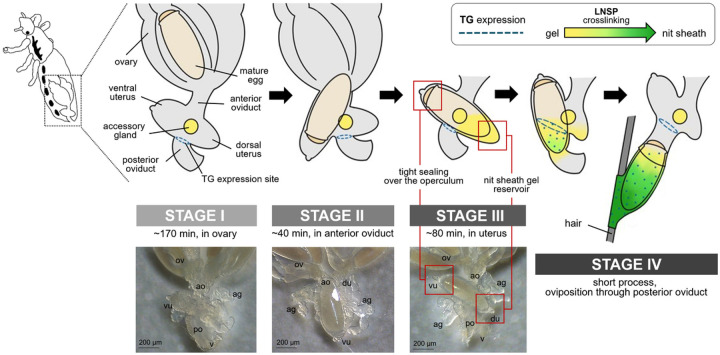
Schematic diagram of the oviposition processes of head louse females. For better view, accessory gland was graphically removed, and connecting area of accessory gland was marked with a yellow circle. Stage I, all mature eggs are still inside the ovary (~170 min); Stage II, mature eggs are ovulated into the anterior oviduct (~40 min). During ovulation, a mature egg first moves to the other side of lateral oviduct, thereby changing its position to upside-down, then to the anterior oviduct; Stage III, mature eggs stay inside the uterus and are coated with nit sheath gel materials in the dorsal end of uterus (~80 min); Stage IV, coated eggs move to the posterior oviduct where crosslinking begins. Actual images corresponding to each stage were provided in the bottom row. ov, ovary; ao, anterior oviduct; vu, ventral end of uterus; du, dorsal end of uterus; ag, accessory gland; po, posterior oviduct; v, vulva.

**Figure 3:**
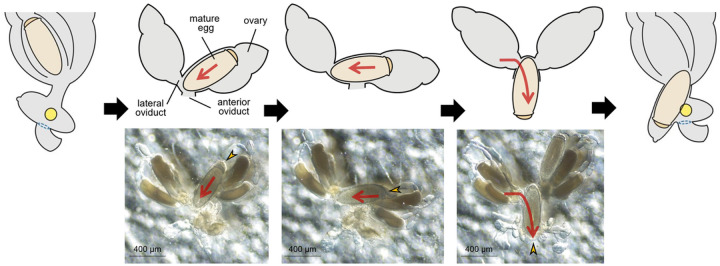
Detailed ovulation processes during the oviposition stages I and II.A mature egg first moves to the other side of lateral oviduct, thereby changing its position to upside-down, then to the anterior oviduct. The red arrows indicate the movement direction of a mature eggs inside the oviduct during ovulation. The yellow arrow heads indicate operculum of an egg.

Detailed microscopic observations revealed that the oviposition processes could be divided into four stages (Fig. 2): Stage I, developing eggs are housed inside the ovary with its operculum side being located toward the head; Stage II, mature eggs are ovulated into the anterior oviduct. During ovulation, a mature egg first moves to the other side of lateral oviduct, thereby changing its position to upside-down, then then enters into the ventral end of uterus with its operculum side moving first (Fig. 3); Stage III, mature eggs move to and stay inside the uterus following ovulation and are coated with nit sheath gel. Once aligned inside the uterus, the mature egg is redirected so that its operculum side toward the head again and its pointed bottom end being positioned toward the vagina; Stage IV, coated eggs move to the posterior oviduct where crosslinking begins. Based on the approximation that one cycle of oviposition takes ~ 290 min (see ‘Examination of oviposition process’ section), the pre-ovulation Stage I, Stage II and Stage III were estimated to take ~ 170 min, ~ 40 min and ~ 80 min, respectively. The last oviposition step (Stage IV) was processed very quickly as described previously [3].

**Figure 4:**
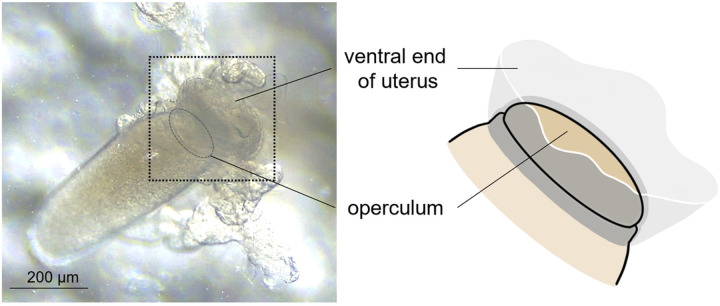
Image of a mature egg with its operculum being attached to the ventral end uterus. Theoperculum stays attached to the ventral end of uterus even after surgically removing the dorsal part of uterus. The ventral uterus turns inside out in this picture.

**Figure 5:**
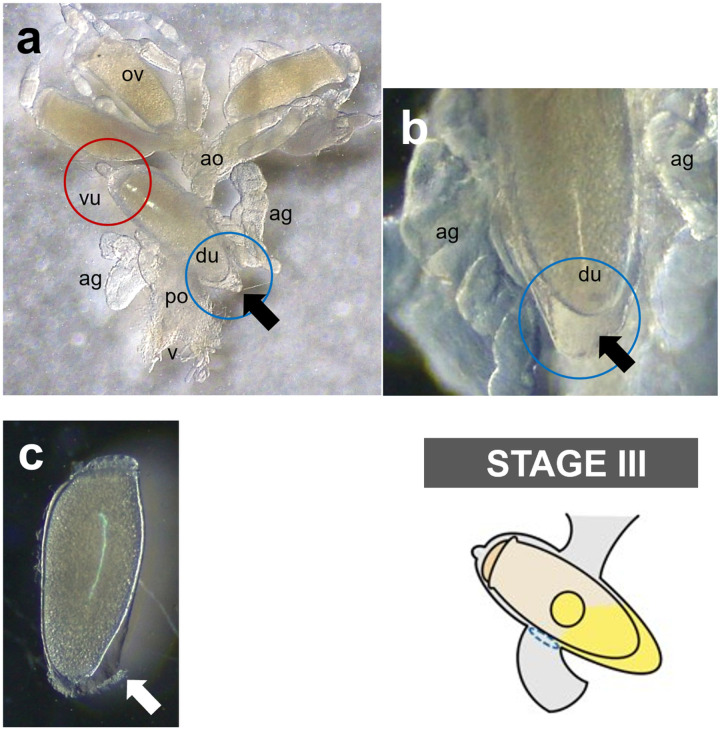
Reproductive system of a female head louse during oviposition. **a, b** A mature egg is aligned inside uterus with the operculum being tightly covered by the ventral end of uterus (see red circle) and with the bottom side being covered by the dorsal end of uterus (see blue circle) during the stage III. Black arrows indicate the nit sheath gel reservoir in the dorsal end of uterus. ov, ovary; ao, anterior oviduct; vu, ventral end of uterus; du, dorsal end of uterus; ag, accessory gland; po, posterior oviduct; v, vulva. **c** An egg dissected out from uterus. A white arrow indicates nit sheath gel coated on the egg.

Interestingly, during Stage III, the mature egg is precisely aligned inside the uterus, with the operculum (top side) being tightly held by the ventral end of uterus and with the bottom side being covered by the dorsal end of uterus (See ‘Stage III’ in Fig. 2). Once held by the ventral end of uterus, a tight connection appeared formed around the circumference of operculum as judged from the observation that the operculum stayed attached to the ventral end of uterus even after surgically removing the dorsal part of uterus (Fig. 4). Thus, like a tight swimming cap, the ventral end of uterus strongly holds the rim of operculum, thereby physically protecting the operculum area from being coated by nit sheath gel and from subsequent crosslinking. In contrast, nit sheath gel was observed inside the dorsal end of uterus, suggesting that the dorsal area of uterus may function as a reservoir for the secreted LNSP1 and LNSP2 (Fig. 5a, b). When an egg was dissected out from uterus, viscous nit sheath gel was isolated with being coated mostly over the bottom of egg (Fig. 5c). This finding suggested that the nit sheath gel is adhesive to the surface of egg but not to the inner wall of uterus.

## Discussion

In the fruit fly, *Drosophila melanogaster,* TG is expressed in hemocytes as either secreted or cytosolic form [8]. The secreted form of TG, which is equivalent to the coagulation factor XIIIa in human blood, is involved in the hemolymph coagulation and hardening by crosslinking various clot components, thereby serving as an immune component [9, 10]. The human head louse TG involved in the crosslinking of nit sheath proteins, however, was expressed only at the opening of posterior oviduct as determined by *in situ* hybridization (Fig. 1c). Further molecular characterization revealed that TG has neither signal sequences at the N-terminus nor putative anchoring site at the C-terminal region. The apparent inhibition of nit sheath solidification by hair-coated GGsTop, an irreversible inhibitor of TG [6], however, suggests that TG is secreted into the lumen of oviduct likely via non-classical secretion pathway [11].

Interestingly, the TG expression site was spatially separated from the ventral end of uterus, which tightly holds the operculum side of mature egg during oviposition, thus likely protecting it from contacting nit sheath gel (Fig. 2). As both LNSP1 and LNSP2 are expressed in the same tissues of the accessory gland and uterus without any physical separation (Fig. 1), confining the crosslinking site to the opening of posterior oviduct should be critical to avoid the uncontrolled crosslinking inside the lumen of accessory gland or the reproductive system. Crosslinking would be initiated from the bottom of egg toward the top during the oviposition process, thereby producing eggs with the operculum side uncoated with nit sheath gel, only when the accessory gland secretion containing LNSP1 and LNSP2 is mixed with TG at the entrance of posterior oviduct (See Stage IV in Fig. 2). The sophisticated mechanism for the precise alignment of a mature egg inside the uterus was revealed, for the first time, through detailed microscopic observation in this study: a mature egg first changes its position to upside-down to make the operculum side first enter the ventral end of uterus in Stage II, and then is repositioned so that its pointed bottom end enters the opening of posterior oviduct, where crosslinking is initiated. In addition to the tight physical protection of the operculum by the ventral end of uterus, additional possible mechanisms may include the presence of protective substances and/or nanostructure inside the ventral side of uterus and/or over the surface of operculum that can impede the nit sheath gel coating and crosslinking. When gel secretion containing TG leaves the vulva, further TG-mediated crosslinking of nit sheath gel continues until complete solidification, as demonstrated by the finding that GGsTop (a TG inhibitor)-coated hair retarded the solidification process of deposited eggs [6]. Following oviposition, the atmospheric oxygen may function as an additional triggering factor for the completion of solidification process [4]. Nevertheless, considering the observation that nit sheath gel was solidified inside the uterus, thus blocking oviposition, when *LNSP2* was knocked down via RNAi [6], the solidification by atmospheric oxygen may not be an essential requirement.

## Conclusions

LNSP1 and LNSP2, major constituents of the nit sheath expressed over the entire area of accessory glands and uterus, become solidified by TG-mediated crosslinking, which occurs at the confined area around the opening of posterior oviduct. Physical separation of the crosslinking site allows selective crosslinking over only the lower part of egg. The precise coordination of the oviposition processes and the nit sheath gel secretion and crosslinking is very crucial for both female and embryo survival, this step can be exploited as a potential target for louse control by disturbing the controlled formation of the egg sheath. In addition, the novel information on the nit sheath forming and egg laying processes in human lice can be expanded to other insect species, facilitating the understanding of hidden functions of the extra egg sheath.

## Abbreviations

LNSP: louse nit sheath protein
TG: transglutaminase
RNAi: RNA interference
PBS: Phosphate buffered saline
SSC: saline-sodium citrate
